# Role of FHOD1 in tumor cells and tumor immune microenvironment

**DOI:** 10.3389/fimmu.2025.1514488

**Published:** 2025-04-29

**Authors:** Yang Yang, Zhanting Kang, Jinlong Cai, Shan Jia, Shangqian Fan, Huifang Zhu

**Affiliations:** Department of Pathology, Xinxiang Medical University, Xinxiang, Henan, China

**Keywords:** FHOD1, tumor immune microenvironment, epithelial-mesenchymal transition, PDL1, PD-1

## Abstract

FHOD 1 (Formin homology 2 domain containing protein 1) is a member of Diaphanous-related formins (DRFs) which contains a GTP-binding domain (GBD), formin homology (FH) 1 and FH 2 domains, a coiled-coil, and a diaphanous-like autoregulatory domain. Studies have shown that FHOD1 can not only regulate intracellular signals in tumor cells but also regulate various components of the tumor microenvironment (TME), such as T cells, B cells, cancer-associated fibroblasts (CAFs), some cytokines. Aberrant expression and dysfunction of the FHOD1 protein play a key role in tumor immunosuppression. Specifically, FHOD1 can impair function of chemokine receptors that are supposed to direct immune cells to localize to the tumor site accurately. As a result of this impairment, immune cells cannot migrate efficiently into TME, thereby impairing their ability to attack tumor cells. In addition, FHOD1 activated signaling pathways within the immune cells abnormally, resulting in their inability to recognize and destroy tumor cells effectively. Therefore, FHOD1 ultimately leads to a state of immunosuppression in TME, providing favorable conditions for the growth and spread of tumor cells. Altogether this review provides an in-depth understanding of the role of FHOD1 in tumor immunosuppression.

## 1 Introduction

Tumors develop within complex tissue microenvironments characterized by dynamic cellar interactions. The initiation of tumorigenesis involves the acquisition of abnormal proliferative capacity through genetic alterations, primarily including activation of proto-oncogenes and inactivation of tumor-suppressor genes. However, a tumor consists not only a group of cancer cells, but also of significant alterations in the surrounding extracellular matrix or tumor microenvironment (TME) (1, 2). These alterations are now recognized as a critical element for tumor development and progression, as well as potential therapeutic targets. Various components of TME, including immune cells and cancer-associated fibroblasts (CAFs), along with diverse cytokines (3), which together impede effective antitumor immunity and promote tumor progression and metastasis.

Formins are multi-domain proteins characterized by the highly conserved formin homology (FH)2 structural domain. This structural domain regulates the nucleation or elongation of actin filaments (4). In addition to the FH2 structural domain, Formins also contains several other structural domains, enabling them to regulate their activation/inactivation state and subcellular localization more finely. For example, some Formins possess specific binding domains at the N-terminal or C-terminal end, which can interact with other molecules (such as signaling molecules, regulatory proteins) to achieve precise regulation of Formin activity (5). In addition, some Formins contain nuclear localization signals or membrane-binding sequences, which enable Formins to function in specific cellular sites, further expanding the diversity of their biological functions. The biochemical properties of individual formins exhibit remarkable diversity and can sometimes be functionally antagonistic: upon activation, they may participate in various actin-related processes, including nucleation, elongation, bundling and capping (6). At the cellular level, individual Formins are involved in the formation of various cell protrusions, and adhesions (7). FHOD1 (Formin homology 2 domain containing protein 1), a 1165-amino acid FH protein, is a potent bundling protein for actin filaments. Unlike most formins, FHOD1 does not elongate actin filaments *in vivo* (8). FHOD1 contains a GTPase binding domain (GBD), FH1 and FH2 domains, a coiled-coil, and a diaphanous-like autoregulatory domain (DAD) (9). In addition, a large number of studies have shown that FHOD 1 plays an important role in tumor cell migration, invasion, and stress fiber formation (10–13). For example, FHOD1 is frequently overexpressed in triple-negative breast cancer, specifically, overexpression of FHOD1 may promote malignant proliferation and metastasis of cancer cells by regulating cytoskeletal stability, cell migration ability, and modulation of various signaling pathways (10). In addition to triple-negative breast cancer, FHOD1 expression has been observed to be significantly uppregulated in glioma cells, a change that correlated strongly with the degree of malignancy and aggressiveness of gliomas, as well as with the prognosis of patients (14). Moreover, FHOD1 upregulates PDL1 expression in tumors through epithelial-mesenchymal transition (EMT) (15), leading to changes in the immune microenvironment. Specifically, FHOD1 regulates the function and activity of T-lymphocytes, cancer-associated fibroblasts (CAF), and B-cells through PDL1, which in turn affects the process of immune escape and immunosuppression in tumors. In this review, considering the pivotal role of FHOD1 in both tumor cells and TME, we aimed to summarize the functions and underlying mechanisms of FHOD1 acts in the communication between tumor cells and TME.

## 2 Effect of FHOD1 in tumor cells

### 2.1 Effect on FHOD1 expression during EMT

**Figure 1:**
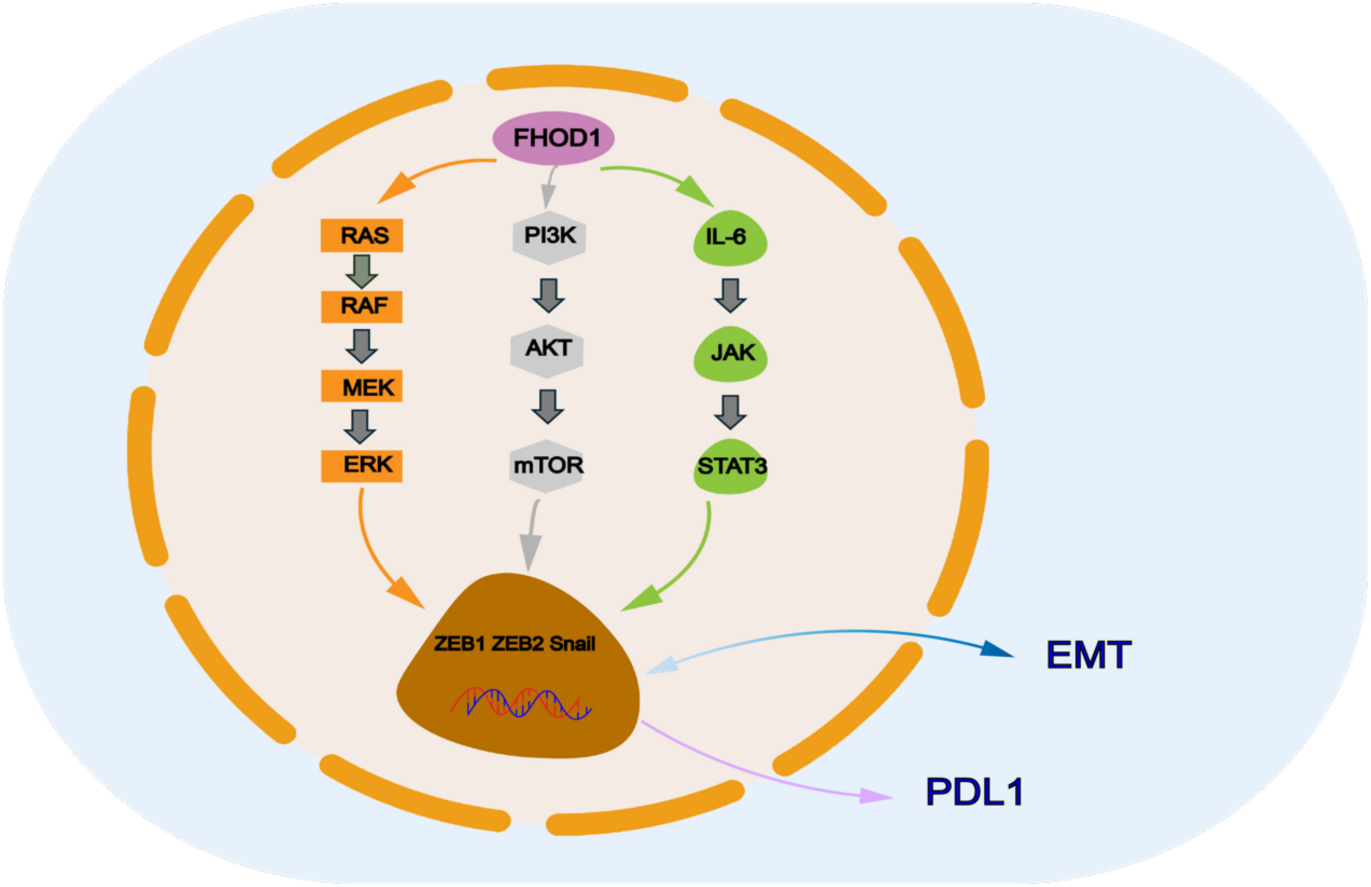
FHOD1 modulates key signaling pathways involved in PDL 1 expression, and downstream signals of these pathways include ZEB and Snail, which are transcription factors of EMT that regulate PDL 1 expression.

Tumor cells acquire invasive and metastasize capabilities through the EMT process (16). This mechanism of cellular plasticity enables malignant cells to remodel their actin cytoskeleton and downregulate cell-cell adhesion proteins. FHOD1 is involved in cytoskeletal remodeling and cell migration in fibroblasts, melanoma cells, and breast cancer cells (15, 17). As actin nucleation- forming are recognized as key regulators of EMT, the expression pattern of FHOD1 in human tissues shows a clear mesenchymal preference, with predominant expression in mesenchymal cells and little expression in epithelial cells. Notably, this expression pattern was significant altered in oral squamous cell carcinoma, where FHOD1 was observed to be upregulated in a PI3K signaling-dependent manner after EMT (**Figure 1**) (13). In EMT-transformed cells, FHOD 1 promotes the development of spindle-shaped morphology and facilitates the formation of mesenchymal F-actin structures. Functional analyses revealed that FHOD1 significantly enhances cell migration and invasion capabilities. Deletion of FHOD 1 impaired the ability of EMT cancer cells to form invasive nuclear peduncles and degrade the extracellular matrix. Based on this finding, we can further explore the potential role of FHOD1 in the tumor microenvironment. EMT is a complex biological process involving changes in cell morphology, adhesion, migration, and invasive capacity, which plays a critical role in tumorigenesis and metastasis (18–20). Therefore, FHOD1 may indirectly affect tumor progression and immune evasion mechanisms by regulating EMT.

### 2.2 FHOD1 promotes PDL1 expression in tumor EMT

Transcription factors play a key role in inducing FHOD1 expression during EMT, which subsequently promotes tumor cell proliferation, migration, and invasion, thereby accelerating tumourigenesis and progression. For example, it has been shown that the EMT phenotype of oral squamous cell is closely associated with elevated expression levels of ZEB1 and ZEB2 (21). Similarly, in cutaneous squamous cell carcinoma, the EMT process is mainly induced by the transcription factor Snail. FHOD1 is significantly upregulated at both transcriptional and protein levels in various EMT cell lines (13). In addition, transcription factors that promote tissue EMT transformation have also been implicated in the regulation of PDL1 expression (22–25). These transcription factors include but are not limited to Snail, Twist, ZEB1, and ZEB2. They directly regulate the transcriptional level of PDL1 by binding to the promoter or enhancer regions of the PDL1 gene. A representative example is the Snail-induced formation of the CCL2/Lcn2 complex, which establishes an immunosuppressive microenvironment that ultimately leads to upregulation of PDL1 expression (26). Thus, the role of FHOD1 in malignant tumors is not limited to the promotion of EMT, it further exacerbates the immune escape phenomenon in tumors by upregulating PDL1 expression (27, 28). This dual regulatory mechanism confers a unique biological function to FHOD1 in cancer (**Figure 1**).

## 3 Role of FHOD1 in tumor immune microenvironment

### 3.1 Relationship between FHOD1 and tumor-infiltrating T lymphocytes

The TME comprises three major components: tumor cells, tumor stroma (inflammatory cells, fibroblasts, and vascular networks), and the surrounding extracellular matrix (29). The non-malignant cells of the TME play an active role in various steps of tumourigenesis (1), significantly influencing tumor behavior and treatment response (3, 30). Within this complex ecosystem,various cells infiltrate the tumor mass and engage in dynamic interactions with tumor cells through both direct cell-to-cell contact and secreted signaling molecules (31). Tumor-infiltrating T lymphocytes play a pivotal role in tumor immunity (32–34), and they exert complex immunomodulatory effects through different subpopulations such as CD4+ T cells and CD8+ T cells (35, 36), as well as anti-tumor pro-inflammatory T cells, immunosuppressive Th2 cells, Th17 cells, and regulatory T cells. These T cells are capable of secreting various immunosuppressive cytokines, such as IL-10 and transforming growth factor β (TGF-β), which further regulate T cell function (37). However, excessive proliferation of tumor cells alters the supply of nutrients and oxygen in TME, forcing T cells to change their metabolic pathways from a dependence on glycolysis to a reliance on fatty acid oxidation(FAO) and oxidative phosphorylation(OXPHOS) to maintain their effector functions. This metabolic adaptation is critical for T cell survival and function in the tumor microenvironment (38). Studies have shown that FHOD1 expression is associated with lymphocyte infiltration in tumor (39). It is noteworthy that human leukocyte antigens are predominantly localized in lymphoid tissues such as the spleen and thymus, as well as in hematopoietic tissues (39). Subsequent investigations have revealed that the substance is overexpressed in human hematological malignancies, especially in non-Hodgkin’s lymphoma and leukemia cell lines. These findings provide important clues for further investigation of its biological functions and potential herapeutic applications (40, 41).

**Figure 2:**
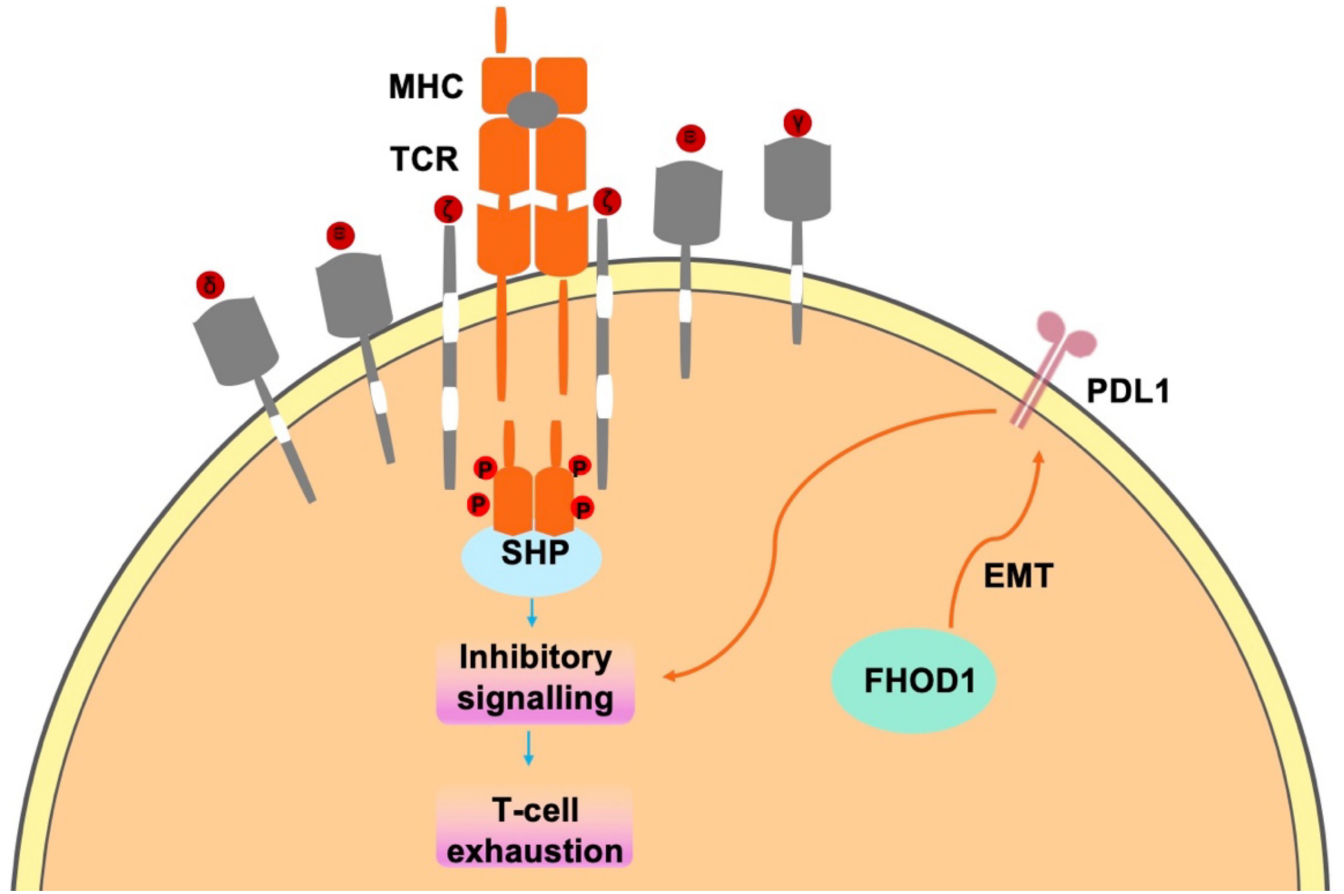
In tumor cells, FHOD1 uses the PD-1/PDL1 pathway to evade immune surveillance mainly by inhibiting t-cell responses in the TME.

In tumor cells, FHOD1 exploits the PD-1/PDL1 pathway to evade immune surveillance, through the suppression of T-cell responses within the TME (42, 43). PD-L1, a key ligand of PD-1, is expressed on the surface of immune cells (44, 45) and tumor cells (31). Upon binding to PD-1, which accumulates near the TCR site, recruits the phosphatase SHP2 to its cytoplasmic domain. Subsequently, SHP2 dephosphorylates proximal TCR signaling molecules, leading to reduced T cell proliferation, reduced cytokine secretion (interferon-γ, IL-2, and tumor necrosis factor-α), altered effector function and reduced survival of activated T cells (**Figure 2**) (31). In summary, FHOD1 enhances the expression level of PDL1 by upregulating its expression during EMT. Therefore, the aberrant expression of PDL1 can be attributed to the upregulation of the oncogenic pathways. These findings elucidate the critical roles of FHOD1 and PDL1 in tumorigenesis, demonstrating their contribution to tumor progression through the regulation of EMT processes (46).

### 3.2 Interaction of FHOD1 and autophagy in CAF

CAFs play a key role in promoting connective tissue proliferation and immunosuppression in TME (47–50), making them potential therapeutic targets for cancer (51–53). Autophagy is a highly regulated, multi-step cellular process that facilitates the transport of cytoplasmic components to lysosomes for degradation and play crucial roles in nutrient recycling and metabolic adaptation (54, 55). FHOD1 is enhanced by the up-regulation of PDL1 expression during EMT, and a large body of literature has shown that upregulation of PDL1 expression causes autophagy in tumor fibroblasts. For example, Xiaozhen Zhang et al. found that in an immunocompetent mouse model, autophagy in CAFs led to up-regulation of PDL1 expression, which resulted tumor immune escape (56). Endo S et al. reported that autophagy of CAFs activates pancreatic stellate cells, promotes the development of pancreatic cancer, and correlates poor prognosis in pancreatic patients cancer (57). In summary, FHOD1 can influence CAF by promoting the up-regulation of PDL1 protein expression (58). This effect was primarily characterized by the enhancement of autophagic activity in CAF cells, a crucial cellular process responsible for intracellular degradation and component recycling. Through autophagy, CAF cells have the potential to help tumor cells evade the surveillance of the immune system, thereby promoting immune escape (53, 59). Immune escape is a key mechanism by which tumor cells evade detection and destruction by the immune system, which is essential for tumor growth and spread. In addition, FHOD1 inhibits TME, including immune cells, extracellular matrix, vascular and other molecular signals, through PDL1-mediated CAF autophagy (60). Inhibition of the TME leads to impaired immune response in the peritumoral milieu, thereby creating a more conducive environment for tumor cell growth and proliferation. Therefore, elucidating the precise role of FHOD1 in this process is important for the development of new tumor therapeutic strategies.

### 3.3 FHOD1 overexpression in tumors stimulates STAT3 activation

The STAT protein family, comprising STAT1 to STAT6, is an important group of transcription factors that play a key role in cell signaling (61–63). Proliferation, differentiation, apoptosis, and inflammation are important biological mechanisms by which STAT proteins regulate cells (64–66). Among the STAT protein family, STAT1, STAT3, and STAT5 are considered to have important roles in cancer cells (67, 68). Among them, STAT1 is involved in anti-tumor immune response, while STAT3 and STAT5 exhibit pro-tumorigenic properties, and their overexpression is closely associated with tumor progression and malignancy (66, 69–71). STAT3 is a key oncogenic transcription factor that is constitutively activated in tumor cells and immune TMEs, serving as a key signaling hub integrating multiple oncogenic signaling pathways (72–74). Abnormally activated STAT3 inhibits apoptosis, induces cell proliferation (75, 76), upregulates matrix metalloproteinase expression, increase matrix stiffness (77), and promotes EMT (75), with the proinflammatory cytokine interleukin-6 (IL-6) being one of the main culprits. It can drive many cancer “hallmarks” by activating the JAK/STAT3 signaling pathway (78). The IL-6/JAK/STAT3 signaling pathway constitutes a self-sustaining regulatory circuit that plays a key role in both cancer development and progression. This molecular cascade can be triggered by chronic inflammation, which is widely recognized as an important risk factor for tumorigenesis (79–83). IL-6 triggers the activation of CAF (84), and activated CAF up-regulates the expression of markers such as α-smooth muscle actin (α-SMA), fibroblast activation protein, platelet-derived growth factor-β, and N-cadherin (85). In addition, activation of FHOD1 protein has been found to be closely associated with the initiation of the STAT3 signaling pathway (86, 87), a process that has attracted much attention in oncology research. Upon activation, FHOD1 induces STAT3 phosphorylation, initiating a downstream signaling cascade that ultimately leads to a significant upregulation of IL-6 expression. As a key pro-inflammatory cytokine in the tumor microenvironment, IL-6 plays a dual role in promoting CAF activation and creating a favorable environment for tumor cell proliferation and metastasis (85).The activation of CAF is a critical step in the process of tumourigenesis and progression. These activated fibroblasts are capable of secreting a variety of growth factors and extracellular matrix proteins, thus providing the necessary support for tumor cells. Mechanistically, FHOD1-mediated STAT3 activation induces IL-6 production, which subsequently triggers CAF activation (88, 89). This cascade of molecular events establishes a microenvironment that promotes tumorigenesis and progression (90–92).

In addition, the STAT3/EMT axis mediates the invasion of a variety of tumors, including colorectal, lung, breast, and brain tumors (93–95). ZEB1/2 protein, TGF-β, Snail, and other EMT regulators are affected by STAT3 signaling (96–98). It has been reported that Snail-induced up-regulation of CSF1R triggers STAT3 signaling while suppressing the expression of miRNA-34a, which promotes the induction of EMT in colorectal carcinogenesis (99). UBE2S up-regulates the expression level of HIF-1α, which stimulates STAT3 signaling, leading to Snail and Twist1 overexpression and inducing EMT (100). we speculate that FHOD1 could trigger a series of downstream events by activating the STAT3 signaling pathway, which in turn promotes cancer cell invasion and metastasis. Specifically,when the STAT3 signaling pathway is activated, a series of gene expression changes are triggered, two transcription factors known to inhibit the expression of epithelial cell markers while promoting the expression of mesenchymal cell markers, which in turn promotes the EMT process (101–103) (**Figure 1**). In addition, the expression of TGF-β and Snail proteins, crucial regulators of the EMT process, was significantly upregulated, thereby greatly enhancing cancer cell migration (79). These findings highlight the role of FHOD1 in driving colorectal cancer progression and metastasis, establishing its key molecular role in tumor progression.

### 3.4 Interaction between FHOD1 and CD21 in human B cells

CD21 is a multifunctional cell surface glycoprotein that is highly expressed in B lymphocytes and follicular dendritic cells (FDCs) (104). However, it is also present in a variety of other cell types.CD21 is a receptor for complement C3dg fragments (105), for CD23 (106), and itself. CD21 consists of an extracellular region consisting of 15–16 short consensus repeat units, a hydrophobic transmembrane region, and an intracellular region of 34 amino acids (107, 108). These three known ligands bind to two N-terminal repeats (106, 107), and crystal structure analysis suggests that they form a highly flexible domain (109). The role of human CD21 in the immune response has been extensively studied and is now well characterized (110, 111). As a key component of the B-cell co-receptor complex, CD21 plays a crucial role in regulating antibody production through multiple mechanisms. These include modulation of B-cell receptor (BCR) signaling through the immune complex (C3d-Ab-Ag), promotion of Fc receptor signaling, and enhancement of B cell memory through the retention of pathogens (including HIV) on FDCs (107, 112–115). The interaction of CD21 and CD23 on B cells is thought to protect B cells from apoptosis (116). However, the function of CD21 on most other cell types remains unclear (117).

**Figure 3:**
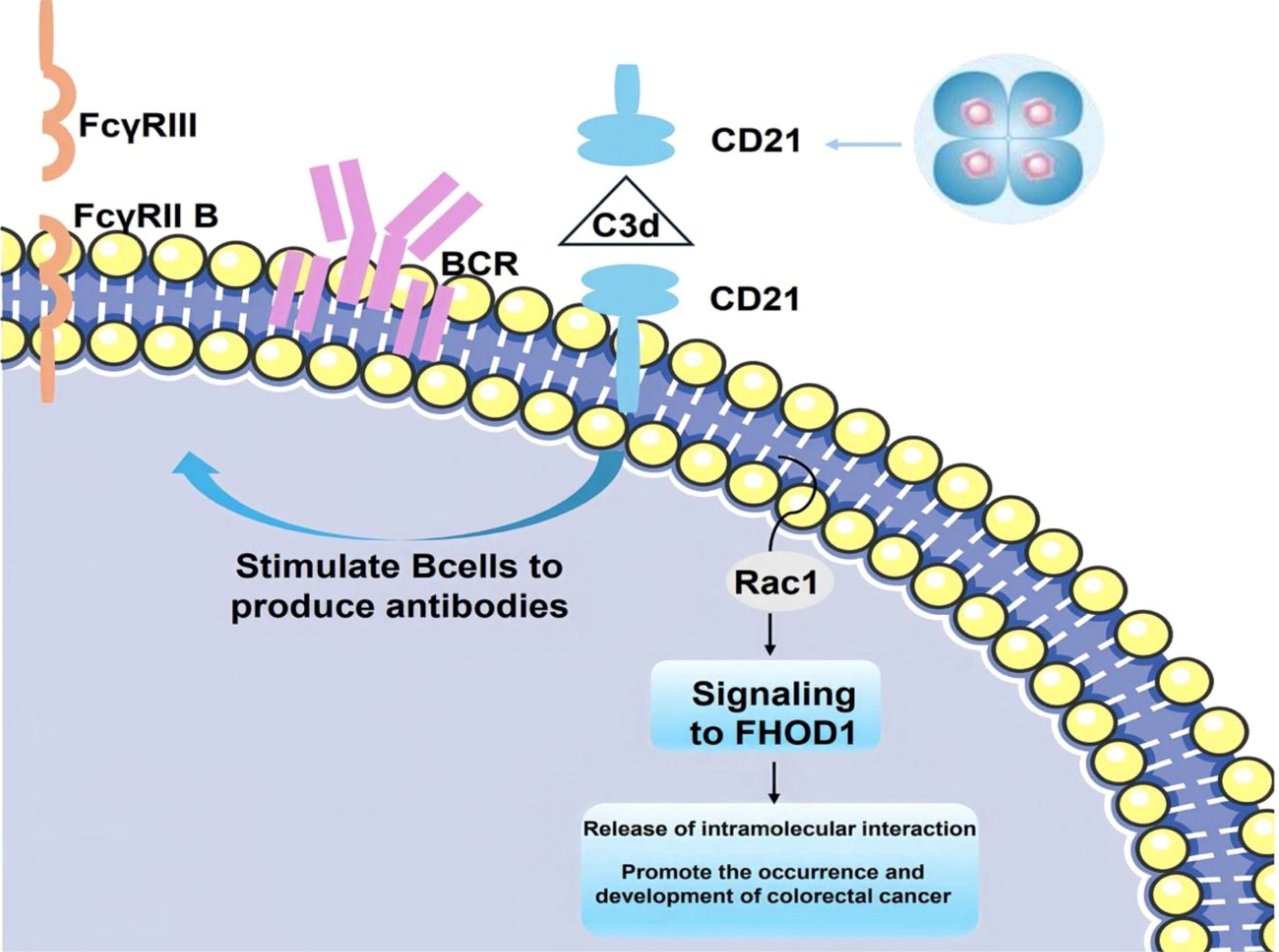
The covalent attachment of activated C3 d to antigen targets the complex to follicular dendritic cells. CD21 stimulates B cells to participate in the regulation of antibody production through BCR signaling mediated by immune complexes (C3d-Ab-Ag) (118). When CD21 is stimulated, the mechanical force generated by Rac1 attracts FHOD1 to the vicinity of CD21, thereby facilitating promoting the development and progression of colorectal cancer.

The interaction of FHOD1 with CD21 in mammalian cells is realized through the C-terminus, a region containing a dad-like structural domain that binds to its own N-terminus through an intramolecular inhibitory interaction. The binding of phosphorylated Rho GTPase to the n-terminal GTPase-binding domain uncouples this interaction. In colorectal cancer cells, stimulation of CD21 triggers the generation of mechanical forces by Rho GTPase and Rac1, which recruit FHOD1 to the vicinity of CD21. This recruitment facilitates colorectal carcinogenesis and tumor progression. Furthermore, FHOD1 plays a pivotal role in CD21-associated cytoskeletal reorganization and the regulation of intracellular signaling pathways (**Figure 3**) (117).

## 4 Conclusion

Forins represent a highly conserved family of actin regulatory proteins that play crucial roles in various tumor cell functions (119), such as cell morphogenesis, cell division, and cell polarity (120). In addition, FHOD1 is one of the most highly expressed human forints detected in a variety of tumor cell lines and tissues, underscoring its fundamental importance in cytoskeletal organization and associated cellular processes (12), FHOD1 was also shown to be able to influence immunochemokines within tumor cells through signaling pathways. Immunochemokines are signaling molecules that attract immune cells to specific locations, and they play a key role in immune surveillance and tumor immune escape. The regulatory function of FHOD1 in modulating the accumulation of these immunochemokines may significantly impair the immune system’s capacity to recognize and eliminate tumor cells, consequently fostering an immunosuppressive tumor microenvironment.This phenomenon of immunosuppression is a strategy for tumor cells to escape the surveillance of the host immune system, allowing them to survive and spread *in vivo*.

In this exhaustive review, we elucidate the pivotal role of FHOD1 protein in tumor biology, with a specific focus on its regulatory mechanism in upregulating PDL1 expression through the EMT pathway (121–123). Our analysis not only reveals the potential mechanism of FHOD1 in tumor cell proliferation and invasion but also demonstrates its importance in tumor microenvironment. Researchers have found that the upregulation of FHOD1 expression is closely related to the invasiveness of tumor cells, which may provide a new target for tumor therapy.

Furthermore, the upregulation of FHOD1 expression in tumor cells in tumor cells is not limited to the promotion of tumor cell proliferation and invasion, but also involves a complex network of signaling pathways that regulate the expression and function of immune molecules. For example, FHOD1 may play an important role in tumor immune escape by regulating certain key signaling molecules, particularly its effect on the expression of the immune checkpoint molecule PD-L1. Increasing evidence suggests that the aberrant expression of FHOD1 is associated with changes in the immune microenvironment of multiple tumor types, which provides new perspectives for understanding the mechanism of tumor immune escape.

In conclusion, the role of FHOD1 in tumorigenesis and development cannot be ignored, as it regulates tumor cell behavior and remodels the immune microenvironment through multiple mechanisms. These findings not only deepen our understanding of tumor biology, but also provide new perspectives and promising therapeutic targets for cancer treatment. With further research, we expect to gain a more comprehensive understanding of the function of FHOD1 in tumor biology, which will help develop innovative and effective therapeutic strategies (124–126).
